# Safe emergency general surgery—ASGBI statement

**DOI:** 10.1093/bjs/znae303

**Published:** 2024-12-10

**Authors:** Hannah Javanmard-Emamghissi, ASGBI EGS Collaborative, Abdullah Ediz, Katherine Brown, Mohamed Aly, Adam Hague, Adrian Harris, Aisha Ehsan, Aiswarya Sukumar, Ajay Belgaumkar, Alexander Phillips, Alistair Myers, Alysha Shetye, Amir Forouzanfar, Amira Said, Andrea Giorga, Andrew Cresswell, Andrew Mitchell, Anjum Arain, Ankur J Shah, Annabelle Williams, Anne Pullyblank, Anthony Thaventhiran, Arun Kochhar, Bala Piramanayagm, Barry Dent, Ben Stubbs, Benjamin Box, Bhavesh Gohil, Chaminda Sellahewa, Chris Thompson, Christian Macutkiewicz, Christopher J Smart, Clare Cheek, Cleo Kenington, Colin Wilson, Constantinos Simillis, Corinne Owers, Damian Mayo, Dan Frith, Daniel Hancu, Darmin Dieac, David Magee, Declan Dunne, Dilip Agarwal, Dimitrios Tsironis, Disha Mehta, Duncan Bew, Elizabeth Elsey, Emma Davies, Eriberto Farinella, Euan McLaughlin, Ewen Griffiths, Ezzat Chohda, Fahed Youssef, Fanourios Georgiades, Farah Khasawneh, Federico Luvisetto, Frances Howse, Gemma Conn, George Reese, George Ryan, William Rea, Lewis Stevens, Giles Bond-Smith, Giuseppe De Santis, Guy Harris, H Tabry, Harjeet Singh Narula, Helen Thomson, Holly Carpenter, Imran Sharieff, Ioannis Gerogiannis, Ish Ahmed, Jansher Khan, Jeremy Corfe, Jessica Chang, Simon Fallis, J G Finch, Jihène El Kafsi, John Evans, John Wayman, Joseph Seager, Judith Salaman, Justin Alberts, Kamal Aryal, Karekin Keshishian, Kathryn Ecott, Katrina Butcher, Keith Chapple, Krish Ravi, Laura Muirhead, Lauren Stroud, Liz Gemmill, Lucy Scott, Lucy Tzouliadis, Luke Bennett, Wilson Cheah, Luke Bishop, Arin Saha, Lynn Fairless, Madan Mohan Palliyil, Malcolm McFall, Mamie Liu, Marianne Hollyman, Mark Cheetham, Maryam Alfa-Wali, Massimo Varcada, Matthew Bedford, Michael Okocha, Michael Okocha, Williamson, Michael White, Minas Baltatzis, Moataz Ewedah, Mohammed Mazen Sadat, Momotaz Sultana, Mostafa Abdel-Halim, Muhammad Harris Siddique, Muhammad Rafaih Iqbal, Muhammad Umair Rashid, Nagarajan Pranesh, Nasira Amtul, Nayaab Abdul Kader, Nebil Behar, Nick Woodcock, Nuha Yassin, Olga Rutka, Owain Jones, Panagiotis Drymousis, Paul Marriott, Paul O’Loughlin, Paul Sutton, Pawan Mathur, Petros Christopoulos, Philip Pucher, Rachel French, Rajarshi Mukherjee, Rajesh Satchidanand, Rebecca Bradley, Renol M Koshy, Richard Guy, Rina George, Rory Callan, Sam Mason, Samreena Riaz, Samuel Lawday, Sarah Duff, Sarah Epton, Sarah Richards, Sarah Wheatstone, Sathyaseelan Arumugam, Sean Cope, Shwetal Dighe, Simon George, Simon Lascelles, Simon Shaw, Spyros Marinos Kouris, Sriram Subramonia, Stephen Chapman, Stephen Odogwu, Stijn van Laarhoven, Sukitha Namal Rupasinghe, Sumit Midya, Sundar Raj Ashok, Tamer Younes, Tania Magro, Tariq Alhammali, Terence Lo, Theophilus Teddy Kojo Anyomih, Thomas Smart, Tim Cook, Toby Hammond, Victor O Alberto, Vittal Rao, Wyn Price, Yuri Hirayama, Chinnappa Thumma Reddy, Chris Payne, David Watt, Dimitrios Damaskos, Gary Nicholson, Himanshu Wadhawan, Hugh Paterson, James Saldanha, Jeyakumar Apollos, Myra McAdam, Lewis Gall, Gemma Scotland, Patrice Forget, George Ramsay, Patrick Collins, Satheesh Yalamarthi, Abdullah Alqallaf, Ahmed Aly, Mrs Dawn Davies, Chris Morris, Duncan Stewart, Gethin Williams, Greg Taylor, Mahmoud Abdeldayem, Majd Al Shamaa, Nicola Harris, Nik Abdullah, Richard Morgan, Simone Sebastiani, Anand Gidwani, Barry McAree, David Mark, Gary Spence, James Patterson, Michael Mullan, Susan Yoong, Michael Van den Bossche, Sheila Clarke

**Affiliations:** Department of Colorectal Surgery, Nottingham University Hospitals NHS Trust, Queens Medical Centre, Derby Road, Nottingham NG7 2UH, UK

The provision of safe emergency general surgical (EGS) care is a global issue^1^ with changing population demographics, incidence of surgical disease and post-pandemic recovery all impacting the delivery of EGS services. Emergency admissions have risen exponentially over the past decade, with ∼750 000 emergency general surgical admissions per year in England alone^2,3^.

In 2014, the National Emergency Laparotomy Audit reported considerable variation in the organization and delivery of care for emergency laparotomy patients with subsequent recommendations to standardize high-quality care in emergency laparotomy^4–6^.

Here, we present results from a unique census of EGS activity defining current practice and variation and a series of further recommendations for adult EGS provision.

The EGS Steering Group of the Association of Surgeons of Great Britain and Ireland (ASGBI) designed a prospective cross-sectional snapshot survey using a modified Delphi method to cover four areas: hospital infrastructure, EGS workload, surgical workforce and resource availability. Data collection took place between September 2022 and May 2023 with ASGBI members and EGS conference delegates invited to participate via email. Responses were received from 177 NHS Trusts, with a response rate of 88% (*Fig. 1*).

**Fig. 1:**
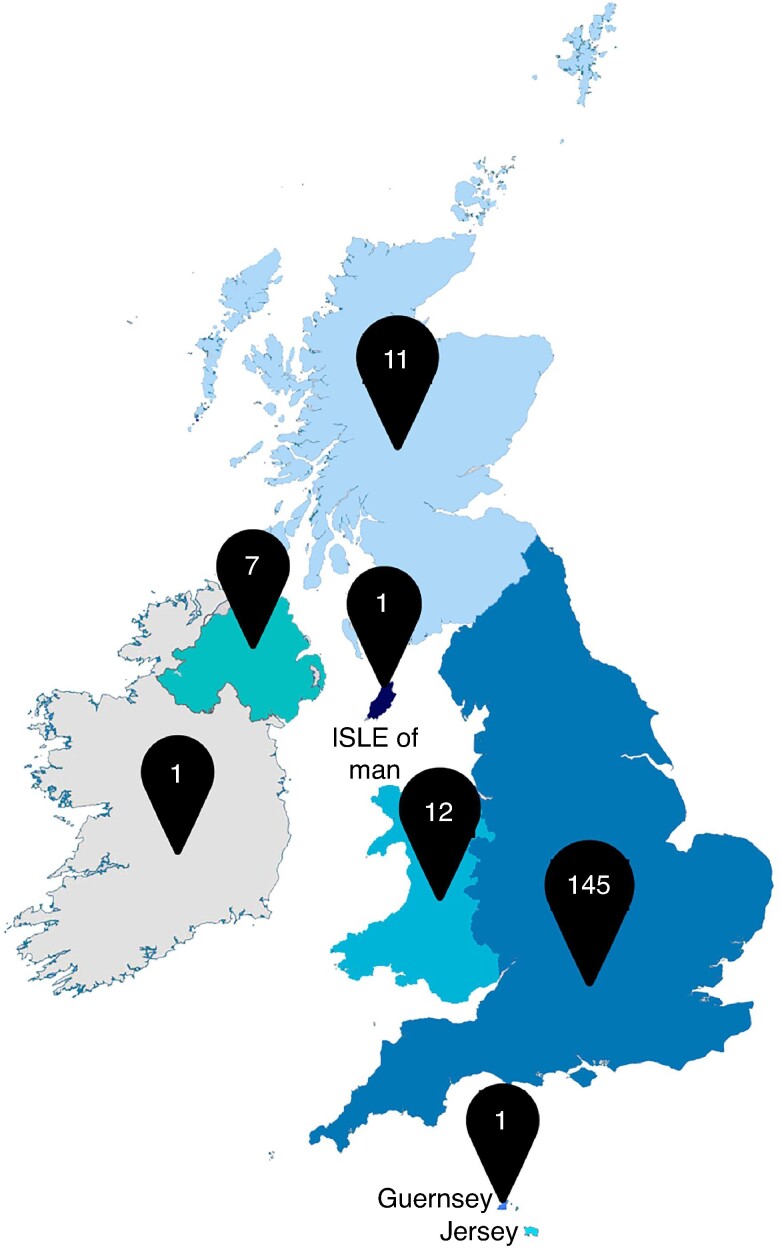
Distribution of responses across the regions of the United Kingdom and Republic of Ireland.

Respondents reported the average number of EGS patient episodes in a 24-hour period, defined as a new patient assessed by the EGS team, irrespective of whether admitted, with activity classified as low (0–10 patient episodes), medium (11–25), high (26–50), and very high (51–75). The majority of sites (86%) reported medium or high activity (*Table 1*).

**Table 1:** Distribution of emergency general surgery patient episodes in 24 hours

Number of emergency general surgery patient episodes	Number of hospital sites	Proportion of total sites included (%)
Low activity	16	9
Medium activity	85	48
High activity	68	38.4
Very high activity	8	4.5

Eighty per cent of hospital sites had a dedicated surgical assessment unit, 77% provided an ambulatory service, often incorporating a consultant-led ‘hot clinic’, and 33% had a dedicated primary care advice line. Although more than 90% of sites reported 24/7 access to an emergency theatre list, less than half of sites had a dedicated EGS theatre, especially those with low and medium EGS activity. All except one site reported 24/7 access to CT yet only 10% of sites had 24/7 access to ultrasonography and only ∼50% centres had 24/7 access to interventional radiology.

Consultant surgeons generally worked separate day/night shifts or a continuous on-call period for either three or four days as a split week. Dedicated EGS surgeons had been appointed in 28% of hospitals, with high- and medium-activity centres reporting the highest proportion (33% and 31% of sites respectively).

Sites reported a median of 11 consultants to cover EGS activity (i.q.r. 8–14) with rotas staffed predominantly by colorectal (47%), upper gastrointestinal (33%), and hepatobiliary surgeons (7%). Specialized emergency general surgeons accounted for 6% of the rota, with breast, vascular, and endocrine surgeons the remaining 8%. In most sites the on-call consultant was responsible for an unselected emergency take with subspecialty on-call availability limited in low-activity sites. The EGS consultant covered paediatric general surgical emergencies in 73% of hospital sites.

## Recommendations for safe and efficient EGS care

Safe staffing levels are essential, defined by EGS admissions and inpatient volume, not hospital size. Patient pathways for all stages of the EGS patient journey should be universally incorporated, especially for the early identification and management of sepsis and the delivery of optimal care for emergency laparotomy patients.

EGS services should be available and contracted 24/7, including for both consultant surgeons and anaesthetists, with access to fully staffed operating theatres. EGS cases should receive appropriate prioritization and without competition with elective care. 24/7 access to cross-sectional imaging and interventional radiology should be available alongside critical care with outreach to deliver protocol-triggered reviews and escalation.

There should be a lead clinician for EGS with allocated contracted time, and training in EGS should ensure required competencies are acquired for completion of training.

Daily input from geriatricians for older patients, irrespective of procedure, should be available, with regular multidisciplinary reviews of processes and patient outcomes for all EGS patients.

## ASGBI EGS collaborative group

### Writing group

Hannah Javanmard-Emamghissi* (UK), Adam Peckham-Cooper (UK), Phill Pearce (UK), Brett Doleman (UK), Susan Yoong (UK), Jared M. Wohlgemut (UK), Arun Chokkalingam (UK), Daniel Lawes (UK), Eriberto Farinella (UK), Haussam Elenin (UK), Peter Judge (UK), Sathasivam Rajeev (UK), Kate Hancorn (UK), Yasser KayyalI (UK), Oliver Ng (UK), David Saunders (UK), Chris Payne (UK), Matt J. Lee (UK), Susan Moug (UK), Sonia Lockwood† (UK), Gillian M. Tierney† (UK)

*First author

†Joint senior authors

### Collaborators

Abdullah Ediz, Katherine Brown, Mohamed Aly (Luton and Dunstable University Hospital, Bedfordshire Hospitals NHS Foundation Trust, Luton, England, UK); Adam Hague (Rotherham District General Hospital, The Rotherham NHS Foundation Trust, Rotherham, England, UK); Adrian Harris (Hinchingbrooke Hospital, North West Anglia NHS Trust, Huntingdon, England, UK); Aisha Ehsan (St Peter’s Hospital, Ashford and St Peter’s NHS Foundation Trust, Chertsey, England, UK); Aiswarya Sukumar (Russells Hall Hospital, Dudley Group of Hospitals NHS Foundation Trust, Dudley, England, UK); Ajay Belgaumkar (East Surrey Hospital, Surrey and Sussex Healthcare NHS Trust, Redhill, England, UK); Alexander Phillips (Royal Victoria Infirmary, The Newcastle upon Tyne NHS Foundation Trust, Newcastle upon Tyne, England, UK); Alistair Myers (Hillingdon Hospital, Hillingdon Hospitals NHS Foundation Trust, Uxbridge, England, UK); Alysha Shetye (Queen’s Hospital, Barking Havering Redbridge University Trust, Romford, England, UK); Amir Forouzanfar (Royal Oldham Hospital, Northern Care Alliance, Oldham, England, UK); Amira Said (Darent Valley Hospital, Dartford and Gravesham NHS Trust, Dartford, England, UK); Andrea Giorga (Harrogate Hospital, Harrogate & District NHS Foundation Trust, Harrogate, England, UK); Andrew Cresswell (Rotherham District General Hospital, The Rotherham NHS Foundation Trust, Rotherham, England, UK); Andrew Mitchell (Darlington Memorial Hospital, County Durham & Darlington NHS Foundation Trust, Darlington, England, UK; University Hospital of North Durham, County Durham & Darlington NHS Foundation Trust, Durham, England, UK); Anjum Arain (North Devon District Hospital, Royal Devon and Exeter NHS Foundation Trust, Barnstaple, England, UK); Ankur J. Shah (William Harvey, East Kent Hospitals University Foundation Trust, Willesborough, England, UK); Annabelle Williams (Milton Keynes University Hospital, Milton Keynes University NHS Foundation Trust, Milton Keynes, England, UK); Anne Pullyblank (Southmead Hospital, North Bristol NHS Trust, Bristol, England, UK); Anthony Thaventhiran (Maidstone Hospital, Maidstone and Tunbridge Wells NHS Trust, Maidstone, England, UK); Arun Kochhar (Tunbridge Wells Hospital, Maidstone and Tunbridge Wells NHS Trust, Royal Tunbridge Wells, England, UK); Bala Piramanayagm (George Eliot Hospital, George Eliot Hospital NHS Trust, Nuneaton, England, UK); Barry Dent (Queen Elizabeth Hospital Gateshead, Gateshead Health NHS Trust, Gateshead, England, UK); Ben Stubbs (Dorset County Hospital, Dorset County Hospital NHS Foundation Trust, Dorchester, England, UK); Benjamin Box (Northumbria Specialist Emergency Hospital, Northumbria Healthcare NHS Foundation Trust, Cramlington, England, UK); Bhavesh Gohil (Newham University Hospital, Barts Health NHS Trust, London, England, UK); Chaminda Sellahewa (Russells Hall Hospital, Dudley Group of Hospitals NHS Foundation Trust, Dudley, England, UK); Chris Thompson (Sandwell General Hospital, Sandwell and West Birmingham NHS Trust, West Bromwich, England, UK); Christian Macutkiewicz (Manchester Royal Infirmary, Manchester University NHS Foundation Trust, Manchester, England, UK); Christopher J. Smart (Macclesfield District General Hospital, East Cheshire NHS Trust, Macclesfield, England, UK); Clare Cheek (County Hospital Hereford, Wye Valley NHS Trust, Hereford, England, UK); Cleo Kenington (St George’s Hospital, St Georges University Hospitals NHS Trust, London, England, UK); Colin Wilson (Freeman Hospital, Newcastle Freeman NHS Trust, Newcastle upon Tyne, England, UK); Constantinos Simillis (Addenbrookes Hospital, Cambridge University Hospitals NHS Foundation Trust, Cambridge, England, UK); Corinne Owers (Blackpool Victoria Hospital, Blackpool Teaching Hospitals NHS Foundation Trust, Blackpool, England, UK); Damian Mayo (Salisbury Hospital, Salisbury NHS Trust, Salisbury, England, UK); Dan Frith (St Mary’s Hospital, Imperial College Healthcare NHS Trust, London, England, UK); Daniel Hancu (Royal Berkshire Hospital, Royal Berkshire NHS Foundation Trust, Reading, England, UK); Darmin Dieac (Pilgrim Hospital, United Lincolnshire Hospitals Trust, Boston, England, UK); David Magee (The Royal Marsden Hospital, The Royal Marsden NHS Foundation Trust, London, England, UK); Declan Dunne (Royal Liverpool Hospital, Liverpool University Hospitals NHS Foundation Trust, Liverpool, England, UK); Dilip Agarwal (North Manchester General Hospital, Manchester University NHS Foundation Trust, Manchester, England, UK); Dimitrios Tsironis (St George’s Hospital, St George’s University Hospitals NHS Trust, London, England, UK); Disha Mehta (Barnet Hospital, Royal Free Hospital London NHS Trust, Chipping Barnet, England, UK); Duncan Bew (King’s College Hospital, Denmark Hill Site, King’s College Hospital, Denmark Hill Site, London, England, UK); Elizabeth Elsey (Queens Medical Centre, Nottingham University Hospitals NHS Trust, Nottingham, England, UK); Emma Davies (Royal Lancaster Infirmary, University Hospitals of Morecambe Bay NHS Trust, Lancaster, England, UK); Eriberto Farinella (Lister Hospital, East and North Hertfordshire NHS Trust, Stevenage, England, UK); Euan McLaughlin (University Hospital Coventry and Warwickshire, University Hospital Coventry and Warwickshire NHS Trust, Coventry, England, UK); Ewen Griffiths (Queen Elizabeth Hospital Birmingham, University Hospitals Birmingham NHS Foundation Trust, Birmingham, England, UK); Ezzat Chohda (Whittington Hospital, Whittington Health NHS Trust, London, England, UK); Fahed Youssef (Ipswich Hospital, East Suffolk and North Essex NHS Foundation Trust, Ipswich, England, UK); Fanourios Georgiades (Bedford Hospital, Bedfordshire Hospitals NHS Foundation Trust, Bedford, England, UK); Farah Khasawneh (Leicester Royal Infirmary, University Hospitals of Leicester NHS Trust, Leicester, England, UK); Federico Luvisetto (Derriford Hospital, University Hospital Plymouth NHS Trust, Plymouth, England, UK); Frances Howse (Southampton General Hospital, University Hospitals Southampton NHS Trust, Southampton, England, UK); Gemma Conn (Broomfield Hospital, Mid & South Essex University Hospital NHS Foundation Trust, Broomfield, England, UK); George Reese (Charing Cross Hospital, Imperial College Healthcare NHS Trust, London, England, UK); George Ryan, William Rea, Lewis Stevens (The Royal London Hospital, Barts Health NHS Trust, London, England, UK); Giles Bond-Smith (John Radcliffe Hospital, Oxford University Hospitals NHS Foundation Trust, Oxford, England, UK); Giuseppe De Santis (Leighton Hospital, Mid Cheshire Hospital NHS Foundation Trust, Crewe, England, UK); Guy Harris (St Richard’s Hospital, University Hospitals Sussex NHS Trust, Chichester, England, UK); H. Tabry (Barnet Hospital, Royal Free Hospital London NHS Trust, Chipping Barnet, England, UK); Harjeet Singh Narula (Chesterfield Royal Hospital, Chesterfield Royal Hospital NHS Foundation Trust, Chesterfield, England, UK); Helen Thomson (Pinderfields Hospital, Mid Yorkshire Hospitals NHS Trust, Wakefield, England, UK); Holly Carpenter (Barnsley Hospital, Barnsley Hospital NHS Foundation Trust, Barnsley, England, UK); Imran Sharieff (Queen Elizabeth Hospital, Lewisham and Greenwich NHS Trust, London, England, UK); Ioannis Gerogiannis (Kingston Hospital, Kingston Hospital NHS Foundation Trust, Kingston, England, UK); Ish Ahmed (University Hospital of North Tees, North Tees and Hartlepool NHS Trust, Stockton-on-Tees, England, UK); Jansher Khan (Arrowe Park Hospital, Wirral University Hospital NHS Foundation Trust, Birkenhead, England, UK); Jeremy Corfe (Norfolk and Norwich University Hospital, Norfolk and Norwich University Hospital NHS Foundation Trust, Norwich, England, UK); Jessica Chang, Simon Fallis (Good Hope Hospital, University of Birmingham NHS Trust, Birmingham, England, UK); J. G. Finch (Northampton General Hospital, Northampton General Hospital NHS Trust, Northampton, England, UK); Jihène El Kafsi (Wexham Park Hospital, Frimley Health NHS Foundation Trust, Slough, England, UK); John Evans (Northampton General Hospital, Northampton General Hospital NHS Trust, Northampton, England, UK); John Wayman (Cumberland Infirmary, North Cumbria Integrated Care, Carlisle, England, UK); Joseph Seager (Russells Hall Hospital, Dudley Group of Hospitals NHS Foundation Trust, Dudley, England, UK); Judith Salaman (Royal Blackburn Hospital, East Lancashire Hospitals NHS Trust, Blackburn, England, UK); Justin Alberts (West Suffolk Hospital, West Suffolk NHS Foundation Trust, Bury St Edmonds, England, UK); Kamal Aryal (James Paget University Hospital, James Paget University Hospitals NHS Foundation Trust, Great Yarmouth, England, UK); Karekin Keshishian (Whipps Cross University Hospital, Barts Health NHS Trust, London, England, UK); Kathryn Ecott (Royal Devon and Exeter Hospital, Royal Devon and Exeter NHS Foundation Trust, Exeter, England, UK); Katrina Butcher (Weston General Hospital, University Hospitals Bristol and Weston NHS Trust, Weston-super-Mare, England, UK); Keith Chapple (Northern General Hospital, Sheffield Teaching Hospitals NHS Trust, Sheffield, England, UK); Krish Ravi (Chesterfield Royal Hospital, Chesterfield Royal Hospital NHS Foundation Trust, Chesterfield, England, UK); Laura Muirhead (West Middlesex University Hospital, Chelse and Westminster Hospital NHS Foundation Trust, Isleworth, England, UK); Lauren Stroud (St Helier Hospital, Epsom and St Helier’s University Hospitals NHS Trust, Sutton, England, UK); Liz Gemmill (King’s Mill Hospital, Sherwood Forest Hospitals NHS Foundation Trust, Sutton-in-Ashfield, England, UK); Lucy Scott (Diana, Princess of Wales Hospital, North Lincolnshire and Goole NHS Foundation Trust, Grimsby, England, UK); Lucy Tzouliadis (Basingstoke Hospital, Hampshire Hospitals NHS Trust, Basingstoke, England, UK); Luke Bennett, Wilson Cheah (Poole Hospital, University Hospitals East Dorset NHS Trust, Poole, England, UK); Luke Bishop, Arin Saha (Huddersfield Royal Infirmary, Calderdale and Huddersfield NHS Foundation Trust, Huddersfield, England, UK); Lynn Fairless (James Cook University Hospital, South Tees NHS Trust, Middlesborough, England, UK); Madan Mohan Palliyil (Stepping Hill Hospital, Stockport NHS Trust, Stockport, England, UK); Malcolm McFall (Worthing Hospital, University Hospitals Sussex NHS Trust, Worthing, England, UK); Mamie Liu (Southend Hospital, Mid & South Essex University Hospital NHS Foundation Trust, Southend-on-Sea, England, UK); Marianne Hollyman (Musgrove Park Hospital, Somerset NHS Foundation Trust, Taunton, England, UK); Mark Cheetham (Princess Royal Hospital, Shrewsbury and Telford NHS Hospital Trust, Telford, England, UK; The Royal Shrewsbury Hospital, Shrewsbury and Telford NHS Hospital Trust, Shrewsbury, England, UK); Maryam Alfa-Wali (St Mary’s Hospital, Imperial College Healthcare NHS Trust, London, England, UK); Massimo Varcada (Royal Free Hospital Hampstead Site, Royal Free Hospital London NHS Trust, London, England, UK); Matthew Bedford (Heartlands Hospital, University Hospitals Birmingham NHS Foundation Trust, Birmingham, England, UK); Michael Okocha (Gloucestershire Royal Hospital, Gloucestershire Hospital NHS Foundation Trust, Gloucester, England, UK); Michael Okocha, Williamson (The Great Western Hospital, Great Western Hospitals NHS Foundation Trust, Swindon, England, UK); Michael White (Royal Bournemouth Hospital, University Hospitals Dorset NHS Foundation Trust, Bournemouth, England, UK); Minas Baltatzis (Salford Royal Foundation Trust, Salford Royal Foundation Trust/Northern Care Alliance, Salford, England, UK); Moataz Ewedah (Queen’s Hospital, Barking Havering Redbridge University Trust, Romford, England, UK); Mohammed Mazen Sadat (Macclesfield District General Hospital, East Cheshire NHS Trust, Macclesfield, England, UK); Momotaz Sultana (King George’s Hospital, Barking Havering Redbridge University Trust, Ilford, England, UK); Mostafa Abdel-Halim (Tameside General Hospital, Tameside and Glossop Integrated Care NHS Foundation Trust, Ashton-under-Lyne, England, UK); Muhammad Harris Siddique (Heartlands Hospital, University Hospitals Birmingham NHS Foundation Trust, Birmingham, England, UK); Muhammad Rafaih Iqbal (Basildon University Hospital, Mid & South Essex University Hospital NHS Foundation Trust, Basildon, England, UK); Muhammad Umair Rashid (Queens Hospital, University Hospitals of Derby and Burton NHS Foundation Trust, Burton, England, UK); Nagarajan Pranesh (Warrington Hospital, Warrington and Halton Teaching Hospitals NHS Trust, Warrington, England, UK); Nasira Amtul (St James’s University Hospital, Leeds Teaching Hospitals Trust, Leeds, England, UK); Nayaab Abdul Kader (Broomfield Hospital, Mid & South Essex University Hospital NHS Foundation Trust, Broomfield, England, UK); Nebil Behar (Chelsea and Westminster Hospital, Chelsea and Westminster Hospital NHS Foundation Trust, London, England, UK); Nick Woodcock (York Hospital, York and Scarborough Teaching Hospitals NHS Foundation Trust, York, England, UK; Scarborough Hospital, York and Scarborough Teaching Hospitals NHS Foundation Trust, Scarborough, England, UK); Nuha Yassin (New Cross Hospital, Royal Wolverhampton NHS Trust, Wolverhampton, England, UK); Olga Rutka (Aintree University Hospital, Liverpool University Hospitals NHS Foundation Trust, Liverpool, England, UK); Owain Jones (Countess of Chester Hospital, Countess of Chester NHS Trust, Chester, England, UK); Panagiotis Drymousis (Ealing Hospital, London North West Healthcare Trust, Southall, England, UK); Paul Marriott (Warwick Hospital, South Warwickshire University NHS Foundation Trust, Warwick, England, UK); Paul O’Loughlin (Queen Elizabeth Hospital Gateshead, Gateshead Health NHS Trust, Gateshead, England, UK); Paul Sutton (The Christie Hospital, The Christie Hospital NHS Trust, Manchester, England, UK); Pawan Mathur (Barnet Hospital, Royal Free Hospital London NHS Trust, Chipping Barnet, England, UK); Petros Christopoulos (Torbay Hospital, Torbay Hospital NHS Trust, Torquay, England, UK); Philip Pucher (Queen Alexandra Hospital, Portsmouth Hospitals University NHS Trust, Portsmouth, England, UK); Rachel French (Queen Elizabeth Hospital Gateshead, Gateshead Health NHS Trust, Gateshead, England, UK); Rajarshi Mukherjee (Aintree University Hospital, Liverpool University Hospitals NHS Foundation Trust, Liverpool, England, UK); Rajesh Satchidanand (Southport and Ormskirk Hospital, Southport and Ormskirk Hospitals NHS Trust, Ormskirk, England, UK); Rebecca Bradley (University Hospital Lewisham, Lewisham and Greenwich NHS Trust, London, England, UK); Renol M. Koshy (Royal Preston Hospital, Lancashire Teaching Hospitals NHS Trust, Preston, England, UK); Richard Guy (Arrowe Park Hospital, Wirral University Hospital NHS Foundation Trust, Birkenhead, England, UK); Rina George (Doncaster Royal Infirmary, Doncaster and Bassetlaw NHS Trust, Doncaster, England, UK); Rory Callan (Northampton General Hospital, Northampton General Hospital NHS Trust, Northampton, England, UK); Sam Mason (Homerton University Hospital, Homerton University Hospital NHS Foundation Trust, London, England, UK); Samreena Riaz (University Hospital of North Durham, County Durham & Darlington NHS Foundation Trust, Durham, England, UK); Samuel Lawday (Cheltenham Hospital, Gloucestershire Hospital NHS Foundation Trust, Cheltenham, England, UK); Sarah Duff (Wythenshawe Hospital, Manchester University NHS Foundation Trust, Manchester, England, UK); Sarah Epton (North Middlesex University Hospital, North Middlesex University Hospital, London, England, UK); Sarah Richards (Royal United Hospital, Royal United Hospital Bath NHS Foundation Trust, Bath, England, UK); Sarah Wheatstone (St Thomas’ Hospital, Guy’s and St Thomas’ NHS Foundation Trust, London, England, UK); Sathyaseelan Arumugam (University Hospital of North Durham, County Durham & Darlington NHS Foundation Trust, Durham, England, UK); Sean Cope (Sunderland Royal Hospital, South Tyneside and Sunderland NHS Foundation Trust, Sunderland, England, UK); Shwetal Dighe (Darent Valley Hospital, Dartford and Gravesham NHS Trust, Dartford, England, UK); Simon George (Royal Cornwall, Royal Cornwall Hospitals NHS Trust, Truro, England, UK); Simon Lascelles (Medway Maritime Hospital, Medway NHS Foundation Trust, Gillingham, England, UK); Simon Shaw (Conquest Hospital, East Sussex Healthcare NHS Trust, Hastings, England, UK); Spyros Marinos Kouris (Stoke Mandeville Hospital, Buckinghamshire Healthcare NHS Trust, Aylesbury, England, UK); Sriram Subramonia (South Tyneside District Hospital, South Tyneside and Sunderland NHS Foundation Trust, South Shields, England, UK); Stephen Chapman (Airedale General Hospital, Airedale NHS Foundation Trust, Keighley, England, UK); Stephen Odogwu (Walsall Manor Hospital, Walsall Healthcare NHS Trust, Walsall, England, UK); Stijn van Laarhoven (Bristol Royal Infirmary, University Hospitals Bristol and Weston NHS Trust, Bristol, England, UK); Sukitha Namal Rupasinghe (Whiston Hospital, St Helens and Knowsley Teaching Hospitals NHS Trust, Whiston, England, UK); Sumit Midya (Frimley Park Hospital, Frimley Health NHS Foundation Trust, Frimley, England, UK); Sundar Raj Ashok (Croydon University Hospital, Croydon Health Services NHS Trust, Thornton Heath, England, UK); Tamer Younes (Queen Elizabeth The Queen Mother Hospital, East Kent Hospitals University Foundation Trust, Kent, England, UK); Tania Magro (Stoke Mandeville Hospital, Buckinghamshire Healthcare NHS Trust, Aylesbury, England, UK); Tariq Alhammali (Kettering General Hospital, Kettering General Hospital Foundation Trust, Kettering, England, UK); Terence Lo (Hull Royal Infirmary, Hull University Teaching Hospitals Trust, Hull, England, UK); Theophilus Teddy Kojo Anyomih (Ipswich Hospital, East Suffolk and North Essex NHS Foundation Trust, Ipswich, England, UK); Thomas Smart (Royal Derby Hospital, University Hospitals of Derby and Burton NHS Foundation Trust, Derby, England, UK); Tim Cook (Gloucestershire Royal Hospital, Gloucestershire Hospital NHS Foundation Trust, Gloucester, England, UK); Toby Hammond (Broomfield Hospital, Mid & South Essex University Hospital NHS Foundation Trust, Broomfield, England, UK); Victor O. Alberto (Croydon University Hospital, Croydon Health Services NHS Trust, Thornton Heath, England, UK); Vittal Rao (University Hospital of North Midlands, University Hospital of North Midlands, Stoke-on-Trent, England, UK); Wyn Price (Royal Bolton Hospital, Bolton NHS Foundation Trust, Bolton, England, UK); Yuri Hirayama (Hinchingbrooke Hospital, North West Anglia NHS Trust, Huntingdon, England, UK); Chinnappa Thumma Reddy (Western General Hospital, NHS Lothian, Edinburgh, Scotland, UK); Chris Payne (Ninewells Hospital, NHS Tayside, Dundee, Scotland, UK); David Watt (University Hospital Crosshouse, NHS Ayrshire & Arran, Crosshouse, Scotland, UK); Dimitrios Damaskos (Royal Infirmary of Edinburgh, NHS Lothian, Edinburgh, Scotland, UK); Gary Nicholson (Queen Elizabeth University Hospital, NHS Greater Glasgow and Clyde, Glasgow, Scotland, UK); Himanshu Wadhawan (Forth Valley Royal Hospital, NHS Forth Valley, Larbert, Scotland, UK); Hugh Paterson (Western General Hospital, NHS Lothian, Edinburgh, Scotland, UK); James Saldanha (University Hospital Hairmyres, NHS Lanarkshire, East Kilbride, Scotland, UK); Jeyakumar Apollos (Dumfries and Galloway Royal Infirmary, NHS Dumfries and Galloway, Cargenbridge, Scotland, UK); Myra McAdam, Lewis Gall, Gemma Scotland (Glasgow Royal Infirmary, NHS Greater Glasgow and Clyde, Glasgow, Scotland, UK); Patrice Forget, George Ramsay (Aberdeen Royal Infirmary, NHS Grampian, Aberdeen, Scotland, UK); Patrick Collins (Dumfries and Galloway Royal Infirmary, NHS Dumfries and Galloway, Cargenbridge, Scotland, UK); Satheesh Yalamarthi (Victoria Hospital, NHS Fife, Kirkcaldy, Scotland, UK); Abdullah Alqallaf (Glangwili General Hospital, Hywel Dda University Health Board, Carmarthen, Wales, UK); Ahmed Aly, Mrs Dawn Davies (Withybush General Hospital, Hywel Dda University Health Board, Haverfordwest, Wales, UK); Chris Morris (University Hospital of Wales, Cardiff and Vale University Health Board, Cardiff, Wales, UK); Duncan Stewart (Wrexham Maelor Hospital, Betsi Cadwaladr University Health Board, Wrexham, Wales, UK); Gethin Williams (Royal Gwent Hospital, Aneurin Bevan Health Board, Newport, Wales, UK); Greg Taylor (Morriston Hospital, Swansea Bay University Health Board, Cwmrhydyceirw, Wales, UK); Mahmoud Abdeldayem (Royal Glamorgan Hospital, Cwm Taf Morgannwg University Health Board, Pontyclun, Wales, UK); Majd Al Shamaa (Prince Charles Hospital, Cwm Taf Morgannwg University Health Board, Merthyr Tydfil, Wales, UK); Nicola Harris (Princess of Wales Hospital, Cwm Taf Morgannwg University Health Board, Bridgend, Wales, UK); Nik Abdullah (Ysbyty Gwynedd Hospital, Betsi Cadwaladr University Health Board, Bangor, Wales, UK); Richard Morgan (Glan Clwyd Hospital, Betsi Cadwaladr University Health Board, Rhyl, Wales, UK); Simone Sebastiani (Bronglais General Hospital, Hywel Dda University Health Board, Aberystwyth, Wales, UK); Anand Gidwani (Altnagelvin Hospital, Western Health and Social Care Trust, Derry/Londonderry, Northern Ireland, UK); Barry McAree (Antrim Area Hospital, Antrim Area Hospital in Northern Trust, Antrim, Northern Ireland, UK); David Mark (Craigavon Area Hospital, Southern NHS Trust, Craigavon, Northern Ireland); Gary Spence (Ulster Hospital, South Eastern Health and Social Care Trust, Belfast, Northern Ireland, UK); James Patterson (Causeway Hospital, Northern Health and Social Care Trust, Coleraine, Northern Ireland, UK); Michael Mullan (South West Acute Hospital, Western Health and Social Care Trust, Enniskillen, Northern Ireland, UK); Susan Yoong (Royal Victoria Hospital, Belfast Health and Social Care Trust, Belfast, Northern Ireland, UK); Michael Van den Bossche (Princess Elizabeth Hospital, Princess Elizabeth Hospital Guernsey, Guernsey, Channel Islands, UK); Sheila Clarke (Noble’s Hospital, Manx Care, Isle of Man, Isle of Man, UK).

## Data Availability

The data underlying this article will be shared on reasonable request to the corresponding author.
